# Complete Genome Sequences of Hepatitis B Virus from North India Using Ion Torrent

**DOI:** 10.1128/genomeA.01065-15

**Published:** 2015-12-10

**Authors:** Shantanu Prakash, Amita Jain, Akanksha Seth, Bhawana Jain

**Affiliations:** Virology Laboratory, Postgraduate Department of Microbiology, King George’s Medical University, Lucknow, Uttar Pradesh, India

## Abstract

Hepatitis B virus (HBV) is among the most common causes of liver cirrhosis. We report the full-genome sequences of seven molecular clones of HBV genotype A, amplified from an HBV-infected North Indian patient. This is probably the first report of the HBV genome sequencing using Ion Torrent from India.

## GENOME ANNOUNCEMENT

Hepatitis B virus (HBV) is an etiological agent of chronic liver disease, including chronic hepatitis, liver cirrhosis, and hepatocellular carcinoma. This poses major health problems worldwide. An estimated 240 million people are chronically infected with hepatitis B (defined as hepatitis B surface antigen positive for at least 6 months), and more than 780,000 people die every year due to cirrhosis and liver cancer (1).

The HBV genome is a partially double-stranded circular DNA molecule of approximately 3,200 bp that encodes four overlapping open reading frames (ORFs) (2). Eight distinct genotypes (A to H) of HBV have been identified, and this classification is based on the distance of the nucleotide sequence from the viral genome of ≥8% (3, 4). HBV genotypes display distinct geographical distributions (5) and impact clinical and antiviral therapy outcomes (6). In broad terms, genotype B is more related to mild liver diseases than genotype C, and genotype D has a less positive prognosis than genotype A (7). Thus, understanding the molecular epidemiology of HBV is critical to improving control and treatment of the infection (8, 9).

Here, we present the full-length human HBV sequences isolated in North India. The virus was detected in serum samples from patients presenting with chronic liver disease. Of all the samples, the complete genome of seven were sequenced by next-generation sequencing (Ion Torrent). Viral DNA was extracted from the patient serum using the Purelink nucleic acid extraction kit (Invitrogen). A full-length genome sequence was obtained by conventional PCR using HBV specific primers and Phusion high-fidelity DNA polymerase. The library was prepared using an Ion Xpress Plus gDNA fragment library preparation kit according to the manufacturer’s protocol (10). Each library was sequenced on an Ion 314 chip after multiplexing with different bar-coded adaptors (Life Technologies). Overlapping sequences were assembled manually with the Ion Torrent server by mapping closely related genomes to establish the whole genome. The mapped file was viewed with the Integrative Genomics Viewer (IGV). The coverage depth of the sequencing varied from 150× to 900×. Phylogeny reconstructions of the whole-genome sequences using the neighbor-joining method demonstrated that the hepatitis B virus isolated from seven North Indian patients clustered with the genotype A reference strain. The whole-genome sequences of hepatitis B virus will help researchers become familiar with HBV strains in North India.

### Nucleotide sequence accession numbers.

The genome sequences are available in GenBank under the accession numbers KM243029, KT151611 to KT151615, and KT151617.
